# Multifocal Head and Neck Neurofibromas with Osseous Abnormalities and Muscular Hypoplasia in a Child with Neurofibromatosis: Type I

**DOI:** 10.1155/2016/3980270

**Published:** 2016-06-12

**Authors:** Rachna Rath, Sheetal Kaur, Shadab Ali Baig, Punyashlok Pati, Sonalisa Sahoo

**Affiliations:** ^1^Department of Oral & Maxillofacial Pathology, SCB Government Dental College & Hospital, Cuttack 753007, India; ^2^Department of Radio Diagnosis, Acharya Harihar Regional Cancer Centre, Cuttack 753007, India; ^3^Department of Oral & Maxillofacial Surgery, SCB Government Dental College & Hospital, Cuttack 753007, India

## Abstract

Neurofibromatosis type 1 (NF1) is a clinically and genetically distinct disease involving both neuroectodermal and mesenchymal derivatives. Orofacial manifestations in NF1 have been documented before but occurrence of multifocal intraosseous (IO) and extraosseous (EO) neurofibromas is rare. The present case highlights the importance of imaging findings in the diagnosis and management of multifocal jaw, infratemporal, and parotid neurofibromas with muscular hypoplasia in an eight-year-old girl with NF1. Apart from orthopantomograms (OPG), three-dimensional computed tomography (3D CT) and cross-sectional reformations were valuable in delineating the extent of the lytic lesion and identifying additional bony deformities of the mandible. Magnetic resonance imaging (MRI) helped to identify the solid nature of the lesion and true extent of the soft tissue mass.

## 1. Introduction

NF1 or von Recklinghausen's disease is a neurocristopathy presenting with certain cutaneous, ophthalmologic, and skeletal manifestations [1]. Head and neck are a common location for NF; however other sites may be involved less frequently including nasal cavity, paranasal sinus, nasopharynx, orbit, larynx, maxilla, and mandible [2]. Intraoral involvement may be in the form of soft tissue neurofibromas, developmental defects, or dental abnormalities.

Neurofibroma (NF) extending to the maxilla and associated with multifocal lesions in the infratemporal fossa, temporomandibular joint, parotid, and external auditory canal is extremely rare. Only one such case has been stated in literature showing multifocal jaw and soft tissue tumors [3]. Management of IO neurofibroma varies depending on age of the patient, tumor location, size, extent, nature (simple or plexiform), presence of functional impairment, and the risk of malignant transformation [4]. It is in this context that advanced imaging tools like CT and MRI become increasingly important, as they aid in establishing the diagnosis, knowing the true extent of the tumor, identifying complications before they become clinically apparent, even in guiding the management as in this case of multifocal neurofibromas in an 8-year-old child with NF1.

## 2. Case Report

**Figure 1:**
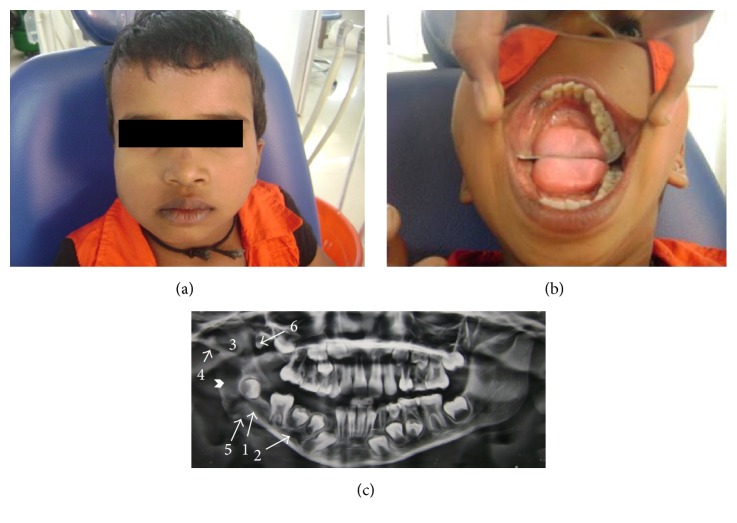
(a) Extraoral frontal view shows swelling on right side of face, (b) intraoral view shows alveolar enlargement and missing teeth on right side, (c) OPG shows intraosseous radiolucency (chevron) with impacted 46, enlarged mandibular canal (1), enlarged mental foramen (2), deep sigmoid notch (3), narrow condyle and pseudoelongation of condylar neck (4), notching of gonial angle (5), and supernumerary tooth in maxilla (6).

An 8-year-old girl referred to the Dental Department presented with right facial asymmetry due to a slow growing painless swelling of 6-month duration. There was periauricular soft tissue swelling and intraoral bony hard enlargement of right mandibular alveolar ridge (Figures 1(a) and 1(b)). A history of antibiotic administration and extraction of the deciduous canine and first and second molar under the assumption of odontogenic infection was elicited.

**Figure 2:**
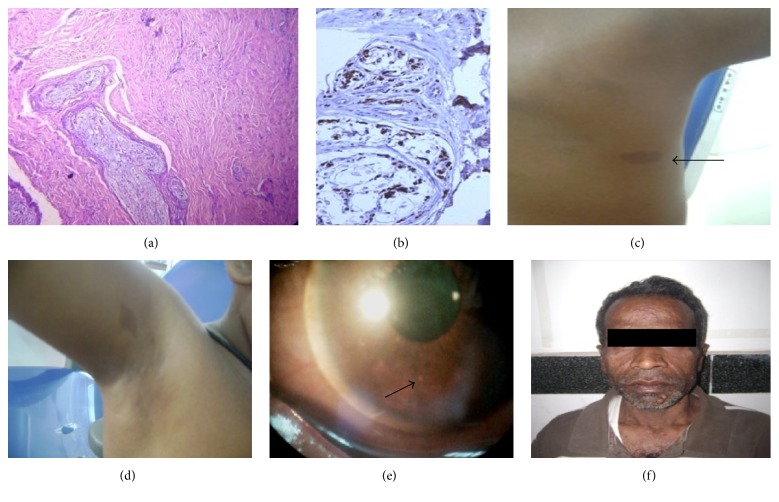
(a) Microsection showing bundles of wavy spindle cells with serpentine nuclei in fascicles (H&E, ×10x), (b) Strong S-100 positivity of tumor cells (×40x), (c) café au lait macule (arrow) in the back, (d) axillary freckle, (e) Lisch nodule (arrow) in slit lamp examination, and (f) father of patient with multiple cutaneous neurofibromas.

OPG showed the presence of a well demarcated radiolucency in the ramus and angle of the mandible. There was mandibular hypoplasia with narrow right condylar process, deepening of the right sigmoid notch, pseudoelongation of the ipsilateral condylar neck, thinner ramus and body, widened inferior alveolar canal, and enlarged mental foramen. Deep notching of gonial angle, narrowing of glenoid fossa, and presence of a supernumerary tooth in right maxillary third molar region were other findings (Figure 1(c)). An incision biopsy from the posterior mandibular lesion which microscopically revealed interlacing bundles of spindle shaped cells with wavy nuclei, delicate collagen fibres, plump fibroblasts, and a strong positive reaction with* S-100* (detection system, HRP polymer) confirmed the diagnosis of simple neurofibroma (Figures 2(a) and 2(b)).

Systemic examination revealed presence of multiple hyperpigmented macules, axillary freckling, and Lisch nodules (Figures 2(c), 2(d), and 2(e)). Patient had short height for her age and macrocephaly but no cutaneous neurofibromas. Examination revealed that her father and grandmother suffered from NF1 (Figure 2(f)). Based on family history and clinical and radiographic findings, NF1 was diagnosed.

**Figure 3:**
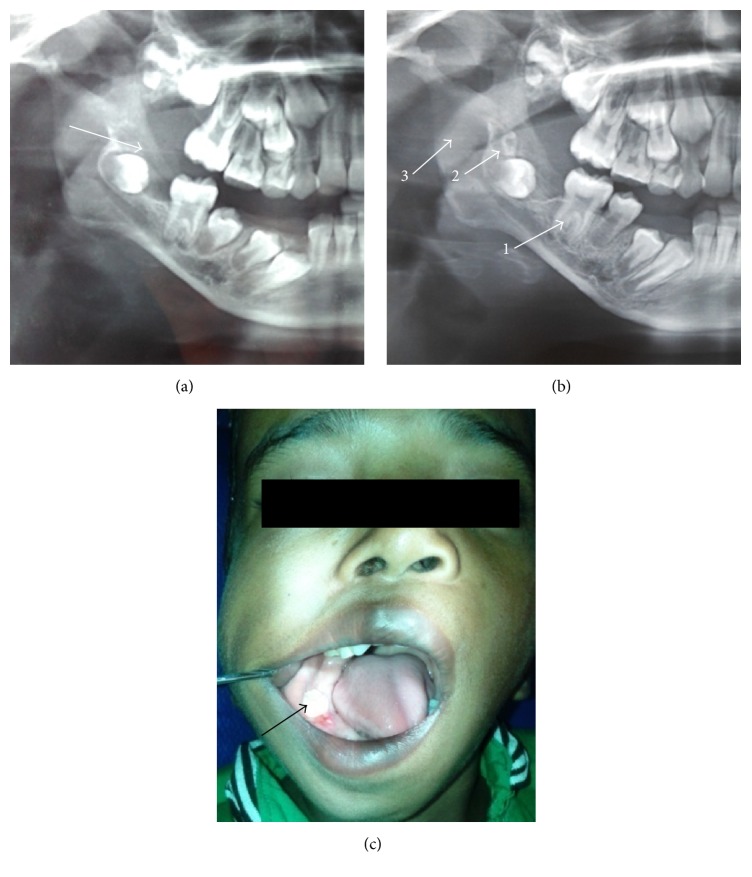
(a) One-year follow-up OPG shows reduced tumor size (arrow), (b) OPG after 1 year and 8 months shows erupted first molar (1), appearance of follicle of third molar (2) and presence of residual tumor (3), and (c) intraoral view after 1 year and 8 months shows fully erupted first molar (arrow).

**Figure 4:**
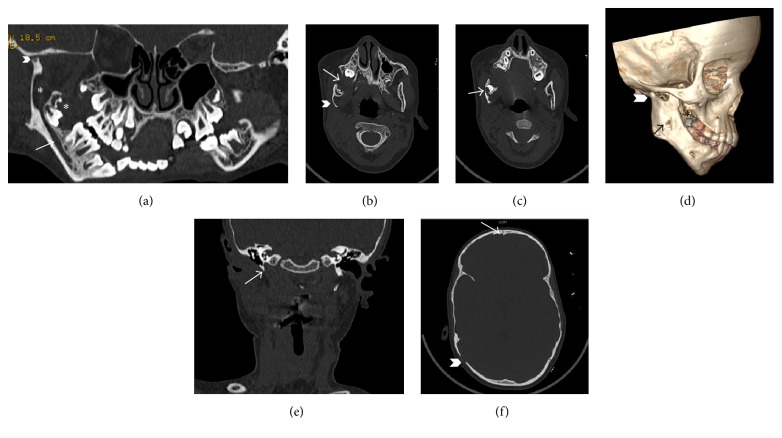
(a)* Panoramic dental scan* depicting enlarged inferior alveolar canal (arrow) with residual tumor mass (asterisk) and hypoplastic condylar process (chevron), (b)* axial CT* in bone window showing the residual mass in alveolar margins of maxilla and mandible (arrow) and concavity of mandibular ramus (chevron), (c)* axial CT scan* showing apparent foramen (arrow) due to lytic erosion of mandibular cortex, (d)* 3D CT scan* image showing hypoplastic condylar process (chevron), apparent foramen (arrow), and tumor in maxillary alveolus (asterisk), (e)* coronal CT* image showing rudimentary styloid process of right side (arrow), and (f)* axial CT* image of sutural diastasis (chevron) and persistent metopic suture (arrow).

**Figure 5:**
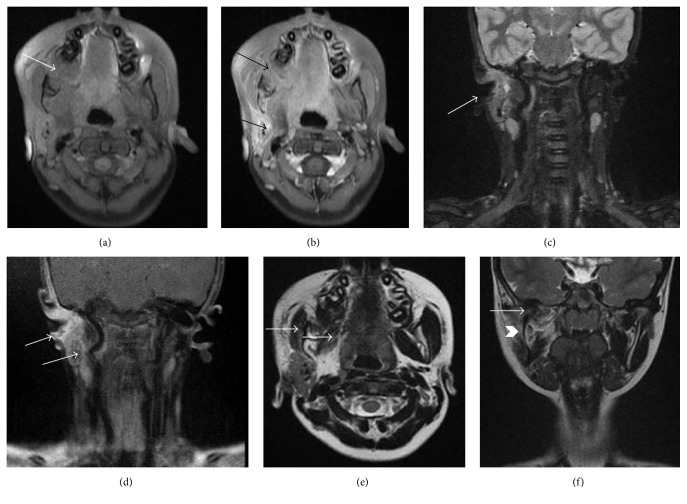
(a)* Precontrast T1W axial* image showing primary mass (arrow) in the alveolar process of maxilla and mandible with bony expansion, (b)* postcontrast T1W fat saturated axial* image showing enhancing intraosseous mass in maxilla and mandible, the parotid, and infratemporal fossa (arrows), (c)* coronal STIR *image showing narrow external auditory canal (arrow), (d)* coronal *postgadolinium image showing mass in infratemporal fossa, the parotid, and external auditory canal (arrows), (e)* axial T2W* image showing hypoplastic pterygoid and masseter muscles (arrows), and (f)* coronal T2W* image showing concave right mandibular ramus (chevron) and hypoplastic condyle with extension of mass into the TMJ (arrow).

As the patient was in an active growth period and had no alarming symptoms, conservative excision of the overlying tumor was done and the first molar was left in situ to erupt. Periodic OPGs revealed that the first molar erupted and the follicle of third molar appeared (Figures 3(a), 3(b), and 3(c)). However, the facial asymmetry and periauricular swelling were persistent. A 3D CT (bright speed 16 slice CT, GE Healthcare) of the mandible was done which revealed an irregular lytic lesion in the alveolar process of right side of mandible extending from lateral incisor to retromolar trigone (Figures 4(a) and 4(b)). An additional small bony defect was seen in the outer cortex of ramus of mandible. This apparent foramen was perhaps due to lytic erosion of the mandibular outer cortex (Figures 4(c) and 4(d)). There was associated lytic expansion of the alveolar margin of right maxilla at level of second molar and floor of right maxillary sinus. Presence of rudimentary supernumerary tooth was also confirmed. Right styloid process was rudimentary (2 mm) and right pterygopalatine fissure was widened (Figure 4(e)). There was persistent metopic suture in the frontal bone. There was also presence of sutural diastasis (12 mm defect of right occipitomastoid suture) (Figure 4(f)). MRI (Signa 1.5 T, Optima GE Healthcare) of the neck with mandible was performed with fat saturated postcontrast spin echo T1 weighted (T1W) and turbo spin echo T2 weighted (T2W) sequence to reevaluate the true extent of the lesion. The lesion had caused expansion of the lateral wall of the right maxillary sinus and alveolar margin of right maxilla and mandible extending from lateral incisor to retromolar area (Figure 5(a)). The lesion was isointense on T1W, hypointense on T2W with mild contrast enhancement on postgadolinium scans suggestive of fibrosis and residual tumor. Ill-defined increased T2W signal intensity and enhancement were seen in right TMJ, right temporal fossa, right infratemporal fossa, right parotid gland, right malar, and preauricular region of face indicating extraosseous infiltration (Figures 5(b) and 5(f)). Right external auditory meatus and canal were hypoplastic with thickened walls (Figures 5(c) and 5(d)). There was thinning and decreased bulk of right masticatory muscles suggesting* hypoplasia* (Figure 5(e)). Mucosal thickening and narrowing of right maxillary antrum and adenoid hypertrophy were associated findings. A repeat ENT evaluation was nonsubstantiative.

Due to lack of radiological evidence of malignant transformation or any functional impairment, patient has been put on a periodic follow-up till date and is being closely monitored.

## 3. Discussion

NF1 is a multisystem disorder that arises from mutation in the NF1 gene located on human chromosome 17q11.2 and encodes a protein neurofibromin [1, 2].

According to the National Institute of Health Consensus Development Conference, the diagnosis of NF1 is based on the presence of two or more of its seven diagnostic criteria [5]. Present case was confirmed as NF1 by presence of family history, numerous café au lait spots in the face and trunk, Lisch nodules, and axillary freckling under the arm.

Intraoral involvement includes the presence of neurofibromas usually affecting soft tissues like tongue, gingiva, palate, cheek, and floor of the mouth [2, 3]. Hypo- or hyperplasia of the maxilla, mandible, zygoma, and TMJ has all been reported [1–4]. Mandibular alterations may be unilateral or bilateral and include enlarged mandibular and mental foramen, widened inferior alveolar canal, branching of mandibular canal, hyperostosis, deepened sigmoid notch, elongated condylar neck, rarefied condylar and coronoid process, medial concavity in mandibular ramus, decreased or flat gonial angle, irregular inferior cortex, and cyst like lesions [1–4, 6]. Maxillary involvement may result in asymmetric maxillary sinuses, expansion of maxillary ridge, hemifacial hyperplasia, and so forth [7]. Neurofibromas of the gingivodentoalveolar complex particularly may cause displacement or impaction of the erupting permanent teeth [4]. Many of the above findings, multifocal neurofibromas in mandible and maxilla and musculoskeletal defects including* hypoplasia of the masticatory muscles* in the present case, were all revealed by CT and MRI. As a recently recognized function of neurofibromin in muscle formation, skeletal muscle deficiency might be a feature of NF1. In fact it is suggested that muscular weakness may contribute to the bone phenotype in NF1 [8]. In 90% children with NF1, NF presents with high frequency of hyperintense signal appearance on T2W and low signal intensity on TIW on MRI, as seen in present case. MRI with gadolinium enhancement is valuable as it offers superior soft tissue contrast and detailed imaging [9].

Neurofibromas of head and neck may arise form cranial nerves like the trigeminal nerve (TGN), uncommonly the facial nerve, glossopharyngeal nerve, or upper cervical nerves [9, 10]. Location of the lesion dictates clinical symptoms and presentation. Upper cervical nerve involvement involves scalp, skull base, posterior auricular area, and so forth and causes massive expansion of these sites [9]. TGN root involvement in posterior fossa would present with intradural tumors, and the gasserian ganglion involvement in middle cranial fossa would present with extra dural mass apart from symptoms of continuous facial pain, facial paresthesia, numbness, and so forth. There would also be associated radiographic signs of erosion of petrous bone, displacement of carotid artery, and so on [11, 12]. Involvement of peripheral branches of TGN is more common. Upper TGN NF involves orbit, upper eyelid, and upper part of face, medial TGN tumor involves mid face and upper alveolus, and lower TGN involves jaws and lower alveolar region [9–11]. In our case due to multifocal involvement of the jaws, preauricular area, parotid, and TMJ and due to osteolytic enlargement of the mandibular canal, there is a possibility that the tumor has originated from medial and lower branches (inferior alveolar nerve) of TGN and facial nerve [10]. However, origin from TGN with extraosseous infiltration is more likely as facial nerve involvement was not identified on CT and MRI. In fact, hypoplastic right external auditory meatus and canal are attributed to developmental defects associated with NF1 [1].

D'Ambrosio et al. in a systematic clinicoradiographic study of 38 NF patients found head and neck manifestations in 92% of the subjects. They objectively defined the radiographic findings and highlighted the periodic monitoring of patients for oral and radiographic development [1]. Sigillo detected orofacial manifestations in all his paediatric cases of NF1 utilising OPG, CT, and MRI [6]. But only one other case of multifocal NF involving the mandible, maxilla, and orbit has been reported till date apart from the present one [3].

Plexiform NF is highly suggestive but not pathognomonic of NF1 [13]. Present case lacked the gross fusiform nerve expansion and “bag of worm” appearance as well as multiple fascicular arrangements of tortuous nerves histopathologically, which is characteristic of plexiform NF [9]. It was in fact a simple disseminated NF with multifocal involvement in the head and neck [4].

Management of NF1 requires periodic monitoring after baseline evaluation and carefully noticing the changes in consecutive visits to rule out onset of complications like malignant transformation that occurs in 6–29% of cases [1–4]. The indications of the latter feature are sudden increase in swelling, paresthesia, pain, and so forth. Radiographic findings of irregular widening of the canal, poorly circumscribed loss of jaw bone, and irregular loss of lamina dura around teeth raise the suspicion of malignancy in NF [4]. Also, plexiform NFs are associated with a higher rate of malignant transformation at 10–15% [9]. None of these alarming findings were present in our case to warrant surgery. In fact, age of patient at time of surgery influences the outcome. Tumors resected before 10 years of age recurred in 60% of cases compared to tumors resected after 10 years of age which showed a 30% recurrence [14]. So, it is prudent to delay surgery as long as possible in asymptomatic pediatric patients. Definitive management in our patient would need a combined surgical, orthodontic, and prosthodontic treatment which has to be delayed till the pubertal growth spurt has ended.

## 4. Conclusion

Periodic close surveillance is of utmost importance in management of a disease with unpredictable natural history and outcome as NF1. Present report of this rare case with multispectral radiological manifestations underlines the importance of imaging surveillance for the same.
